# Potential Usefulness of the Kampo Medicine *Yokukansan*, Containing *Uncaria* Hook, for Paediatric Emotional and Behavioural Disorders: A Case Series

**DOI:** 10.1155/2013/502726

**Published:** 2013-09-24

**Authors:** Yoshiyuki Tanaka, Takeshi Sakiyama

**Affiliations:** ^1^Comfo Garden Clinic, 3-2 Kawada-cho, Shinjyuku-ku, Tokyo 162-0054, Japan; ^2^Terutane Yamada Memorial Shibuya Clinic, 2-10-7 Dougenzaka, Shibuya-ku, Tokyo 150-0043, Japan

## Abstract

*Background*. Paediatric emotional and behavioural disorders (EBD) are relatively common diseases. Although nonpharmacologic and pharmacologic treatments are utilized in these cases, it is sometimes difficult to manage the symptoms of EBD. Historically, *Uncaria* hook has been used for treating nighttime crying and convulsions in children. Recent clinical studies have demonstrated that the Kampo medicine *Yokukansan* (YKS), which contains *Uncaria* hook, is efficacious for behaviour disorders in Alzheimer's disease patients. Herein, we investigated the clinical efficacy and safety of YKS in a series of cases with paediatric EBD. *Patients and Methods*. We retrospectively reviewed all paediatric patients who sought Japanese Kampo therapy at our outpatient clinics between April 1, 2012, and April 30, 2013; we selected patients who were diagnosed with paediatric EBD and were treated with YKS. *Results*. After screening all candidates, 3 patients were eligible for this analysis. Their average age was 11.6 years (range 10–13 years). All 3 patients responded very well to YKS within 1 month. No drug-related adverse events were observed during the course of YKS treatment. *Conclusion*. *Yokukansan* may be efficacious for paediatric EBD. We believe these results warrant further evaluation of the clinical efficacy and safety of *Yokukansan* for paediatric EBD in a carefully designed, double-blind, randomized clinical study.

## 1. Introduction

Paediatric emotional and behavioural disorders (EBD) are relatively common diseases. Although there is no clear definition of EBD, attention deficit hyperactivity disorder (ADHD), conduct disorder, and autism are considered to be included in EBD. For example, the incidence of physician-diagnosed ADHD in children aged 5 to 11 years in Southern California was 3.1% in 2010 [1]. In fact, a relationship exists between ADHD and schoolteachers' input, as, in many cases, it is a teacher, not a physician, who diagnoses a student with ADHD. Once diagnosed, nonpharmacologic treatments, such as behavioural modification, and pharmacologic treatments, such as administration of stimulants, are commonly used as standard therapies. 

*Uncaria* hook (UH) is the hook or the hook-bearing stem of *Uncaria rhynchophylla* Miquel, *Uncaria sinensis* Haviland, or *Uncaria macrophylla* Wallich (*Rubiaceae*). This drug has been empirically used for a long time to treat hypertension-related symptoms, such as headache and dizziness, and central nervous system related symptoms, such as seizure and epilepsy. The quality of UH used for therapeutic purposes is strictly defined by the Japanese Pharmacopoeia (JP) [2]. The JP specifies the details regarding UH, in particular, its identification, loss upon drying, total ash, extract content, and assays, including the methods, operating conditions, and system suitability. According to these regulations, UH must contain not less than 0.03% of total alkaloids (rhynchophylline and hirsutine), as calculated on a dried basis. The major indole alkaloids that comprise UH include rhynchophylline, isorhynchophylline, corynoxeine, isocorynoxeine, hirsuteine, hirsutine, and geissoschizine methyl and have been demonstrated to possess vasodilative effects [3, 4]. Geissoschizine methyl ether is a potent serotonin-1A receptor agonist [5–7] and may play an important role in the therapeutic efficacy of UH for various physiological and neurological disorders. In Japan, one of the most popular Kampo formulas containing UH is *Yokukansan *(YKS). YKS consists of 7 ingredients, namely, *Atractylodis lanceae rhizoma, Poria, Cnidii rhizoma, Radix Angelicae, Radix Bupleuri, Radix Glycyrrhizae*, and *Uncaria* hook. Several clinical studies have been conducted regarding the behavioural and psychological symptoms of dementia (BPSD) in patients with dementia [8–11]. A systematic review of these studies showed the beneficial effects of YKS on the Neuropsychiatric Inventory (NPI) and Activity of Daily Living (ADL) scores in patients with dementia [12]. Therefore, many Japanese physicians are now commonly using YKS together with Western medicines such as donepezil and memantine for the treatment of dementia. The first description of YKS is considered to be in the classical Chinese paediatric textbook entitled *Bao-Ying-She-Yao*, written in the 16th century, and YKS was introduced in this textbook for paediatric convulsions, nighttime crying, and insomnia. Some Japanese paediatricians empirically use YKS for paediatric EBD and believe that it may be efficacious for that condition. Hence, we retrospectively investigated a series of patients with paediatric EBD who were treated with YKS.

## 2. Patients and Methods

### 2.1. Patients

All paediatric patients who visited our clinics between April 1, 2012, and April 30, 2013, were screened and selected based on following criteria: (a) the diagnosis met the criteria of EBD in ICD-10; (b) the patient continuously used YKS; and (c) the patients were under 18 years of age. Any patient who took Western drugs with YKS was excluded from this investigation. The diagnoses were reevaluated based on information from their mothers and their schoolteachers. 

### 2.2. Evaluation of Efficacy and Safety

 We identified 3 patients (2 ADHD cases, ICD-10 F90, and 1 school nonattendance case who had physical symptoms derived from emotional and behaviour factors, ICD-10 F54 and ICD-10 F98) who were consecutively treated with YKS. We assessed the treatment outcomes and safety based on periodic feedback from the patients' mothers and schoolteachers. 

### 2.3. Dose and Regimen

 All 3 patients were administered ethical YKS manufactured by Tsumura (TJ-54, Tsumura & Co, Tokyo, Japan), with a starting dose of 5 g per day and a maximum dose of 7.5 g per day. *Shokenchuto* or *ogikenchuto* was also administered with YKS to all 3 patients, as the sweet taste of either kenchuto could increase patient compliance with YKS but would not affect the treatment of paediatric EBD.

## 3. Results

Only three patients met the criteria during the observation period. The patients' average age was 11.6 years (range, 10–13 years). All 3 patients responded very well to YKS, and the average duration between the YKS prescription start date and the response onset was 16.3 days (range 14–21 days). 

Patient 1 was a 13-year-old boy who used to have psychogenic fever. After entering junior high school, he often complained of various symptoms such as abdominal pain and fever prior to going to school. Six months before visiting our clinic, he could not attend any classes. We believed his psychological factors were affecting his physical condition and that his symptoms met the criteria of ICD-10 F54; psychological and behavioural factors that are associated with disorders or diseases are classified elsewhere and are also categorized into ICD-10 F98, other behavioural and emotional disorders, with onset usually occurring in childhood and adolescence. At his first visit to our clinic, he was irritable and restless. Upon physical examination, the patient was thin and had a dark-purplish skin colour around his eyes, a red-purplish tongue, and dilated sublingual veins. His pulse was weak, and he was hypersensitive to touch and had abdominal muscle contractions, as well as subcostal stiffness in his abdomen. YKS and *ogikenchuto* were prescribed. Twenty-one days after starting Kampo treatment, he was able to attend classes 3 days a week; on the 96th day, he had an almost normal school life. The YKS administration was terminated on the 114th day due to the patient's will. No YKS-related adverse reactions were observed throughout the treatment course.

Patient 2 was a 10-year-old boy who was diagnosed with ADHD around the time he entered elementary school. His excessive activity and lack of persistence in cognitively involved activities led to the patient having difficulty attending school, even in a special class. His symptoms met the criteria of ICD-10 F90, hyperkinetic disorder. Although he tried to use stimulants, he discontinued the agents because of drug-induced diarrhoea. His mother brought him to our clinic for Kampo treatment. At his first visit to our clinic, he looked thin and was restless. A rose-pink tongue and dilated sublingual veins were observed. His pulse was weak, and he had hypersensitivity to touch and abdominal muscle contraction, as well as subcostal stiffness in his abdomen. YKS and *shokenchuto* were prescribed. Forty days after beginning YKS, the frequency of his excessive activity was found to have reduced; 57 days later, his behavioural problems had almost disappeared. The patient was still receiving YKS at the time of writing this report. No YKS-related adverse reactions had been observed throughout the treatment course.

Patient 3 was a 12-year-old boy who had excessive activity, lack of persistence in cognitively involved activities, restlessness, and impulsiveness. His behavioural abnormality has been gradually increasing throughout elementary school. Six months prior to visiting our clinic, his teacher recommended that he receive medical intervention to continue school. All his behavioural abnormalities met the criteria of ICD-10 F90, hyperkinetic disorder. His mother wanted him to try the Kampo treatment prior to starting standard therapy, so she brought him to our clinic. At his first visit to our clinic, he was talkative and restless but had normal stature. His pulse was weak. Mild abdominal muscle contraction and subcostal stiffness in his abdomen were observed. YKS and *shokenchuto* were prescribed. Forty days after beginning YKS, the frequency of his excessive activity had reduced; 119 days later, his behavioural problems had almost resolved. The patient was still receiving YKS at the time of writing this report. No YKS-related adverse reaction had been observed throughout his treatment course.

## 4. Discussion

EBD represent a broad category that is commonly used in educational settings for children and adolescents to group a range of more specific perceived difficulties. Both the general definitions and the concrete diagnosis of EBD may be controversial, as the observed behaviour may depend on many factors. ICD-10 elaborates on the diagnostic criteria for EBD. Patients 2 and 3 met the criteria of EBD in ICD-10. However, the first patient's diagnosis might be considered controversial as to whether his symptoms met the criteria of EBD in ICD-10. He did not have any hyperactivity but did have some anxiety while attending school, and his physical problems appeared to be related to his behavioural and emotional factors. Therefore, we categorized him as having EBD and included him in this investigation. 

Based on our literature search, we could not find any predesigned clinical study that included a randomized controlled study with YKS for paediatric EBD. Recently, Miyaoka et al. reported the efficacy and safety of YKS in pervasive developmental disorders and Asperger's disorder by conducting a 12-week prospective, open-label study with 40 subjects aged 8 to 40 years [13]. Interestingly, as this report demonstrated that 36 out of the 40 patients responded to YKS during only a 12-week observation [8], it may be understandable that all of our cases also responded to YKS and showed responses within 1 month. Thus, from a clinical viewpoint, a 1- or 2-month observation period may be enough to judge if a patient may respond to YKS. Moreover, a relatively short observation period may be sufficient to demonstrate the efficacy of YKS if a randomized controlled trial were to be designed.

 In Japan, during the 18th century, Dr. Tokaku Wada discovered that YKS was widely applicable for emotional disorders, not only in the paediatric population but also in the adult population. Based on the clinical experiences reported in Japan over the last 300 years, to date, some clinical manifestations have been targeted by Kampo specialists for a YKS prescription. These manifestations are as follows: (a) feeling of anger, irritability, or both; (b) abdominal rectus muscle contraction; (c) pulsation in the upper abdomen; and (d) subcostal stiffness. All of our cases had abdominal rectus muscle contraction and subcostal stiffness. In Japanese Kampo medicine, this set of manifestations is called *sho*, and a Kampo specialist utilizes* sho* to select a particular formulation. However, the process of incorporating *sho* into a study design for a randomized controlled trial would be a significant issue in establishing scientific evidence for the therapeutic efficacy of YKS.

 The pharmacological mechanisms of YKS for EBD are still unknown. However, recent basic research showed that YKS acts agonistically on serotonin-1A (5-HT_1A_) or serotonin-2A (5-HT_2A_) receptors, dopamine 2 receptors, or both [14]. Furthermore, an in vitro binding study demonstrated that geissoschizine methyl ether, an alkaloid in *Uncaria* hook and a galencial constituent of YKS, binds agonistically to the 5-HT_1A_ and dopamine 2 (D_2_) receptors [15]. Because ADHD has been associated with low levels of dopamine and norepinephrine, an increase in the synaptic concentrations of both norepinephrine and dopamine is a key step for the treatment of ADHD, and the dopamine signal can be significantly enhanced with an agonist or a partial agonist of the 5-HT_1A_ autoreceptors [16–19]. Therefore, the therapeutic efficacy and pharmacological mechanism of YKS might involve an increase in the synaptic concentrations of norepinephrine and dopamine.

## 5. Conclusion

This study provides quite limited evidence for the potential usefulness of YKS due to the limited number of patients. However, considering the excellent safety profile and potential therapeutic benefit of YKS, further investigation, including a carefully designed randomized controlled trial, would be valuable for the treatment of EBD.
